# Mukbang viewership and body image among youngsters: a third-person effect perspective

**DOI:** 10.3389/fpsyg.2026.1776473

**Published:** 2026-08-07

**Authors:** Habin Noh, Xiaojing Li

**Affiliations:** School of Media & Communication, Shanghai Jiao Tong University, Shanghai, China

**Keywords:** body image, mukbang, social media, third-person effect, young adults

## Abstract

Mukbang, a form of digital food entertainment, has become increasingly prominent in contemporary media environments. While prior research has focused on viewing motivations, less attention has been paid to how mukbang exposure relates to body-image evaluations and perceived media influence. Guided by the Third-Person Effect (TPE) framework, this study examined the relationships among mukbang viewing, perceptual gap (self–other discrepancy in perceived media influence), and body-image evaluations among Chinese young adults. A cross-sectional online survey was conducted with 396 participants aged 18–30 who reported prior experience with mukbang content. The results indicated a self–other perceptual discrepancy in the perceived magnitude of mukbang’s influence, with participants perceiving this influence to be greater for others than for themselves. Mukbang viewing was positively associated with body-image evaluations, and this relationship was partially mediated by perceptual gap. In contrast, social distance did not significantly moderate the association between perceptual gap and body-image evaluations. These findings suggest that perceptual discrepancy may function as a cognitive mechanism linking digital media exposure to self-evaluative outcomes, extending the TPE framework to entertainment-oriented food content. However, the results should be interpreted with caution due to the use of non-probability sampling, cross-sectional data, and self-reported measures. Overall, this study highlights the role of indirect cognitive processes in shaping body-image evaluations within algorithm-driven media environments.

## 1 Introduction

China’s digital media landscape has expanded at an unprecedented pace over the past decade, driven by rapid technological development, nationwide mobile connectivity, and the widespread adoption of algorithmic content distribution systems. By 2025, China reported 1.108 billion Internet users, with 99.7 percent accessing online services primarily via mobile devices and 95.5 percent engaging with short-form video platforms (CNNIC, 2025). This media ecosystem is characterized by highly personalized, algorithm-driven recommendation systems that shape how users encounter and consume digital content. National analyses further indicate that short-video use has become deeply embedded in everyday routines, underscoring the centrality of platforms such as Douyin, Kuaishou, and Bilibili in contemporary media life (Liao, 2024).

Young adults aged 18 to 30 represent one of the most active user groups within this environment. This life stage is marked by heightened sensitivity to social comparison, identity formation, and health-related self-regulation, which may increase responsiveness to digitally mediated cues. In this study, the term “young adults” refers to individuals within this age range.

Within this immersive media environment, digital food entertainment has grown rapidly. Mukbang, which originated in South Korea, has become a widely consumed and culturally distinctive form of online food content across East Asia, including China. Mukbang videos typically feature content creators consuming large quantities of food while engaging viewers through conversation or real-time interaction. Prior research characterizes mukbang as a multisensory media practice that integrates visual stimulation, emotional intimacy, and parasocial engagement (Kim and Lee, 2019). Its appeal extends beyond entertainment and is associated with motivations such as companionship, stress relief, vicarious eating, and emotional regulation, particularly among younger audiences in China (Xiao et al., 2024).

At the same time, algorithmic recommendation systems and platform commercialization have accelerated the circulation of mukbang, embedding it in the everyday media routines of Chinese audiences. Growing empirical evidence has identified health and behavioral risks associated with mukbang’s high-intensity eating displays. Frequent exposure has been linked to unhealthy behavioral imitation, increased overeating tendencies, and symptoms of disordered eating among viewers (Kircaburun et al., 2021b; Park et al., 2024).

Taken together, existing evidence indicates that mukbang functions not only as entertainment but also as a culturally embedded media practice shaped by emotional, social, and health-related dynamics. Its growing visibility intersects with increasing public health concerns related to body image among young adults. Body image refers to the cognitive, affective, and evaluative processes through which individuals perceive and interpret their physical appearance (Cash and Smolak, 2019). A substantial body of research demonstrates that repeated exposure to appearance-focused digital environments can heighten appearance anxiety and body dissatisfaction (Holland and Tiggemann, 2016). Similar patterns have been observed within China’s highly visual social media ecosystem, where appearance comparison and influencer-driven aesthetics play a prominent role. Recent evidence further shows that social media–based appearance comparison is strongly associated with body shame and body dissatisfaction among Chinese youth and young adults (Wang et al., 2025).

While prior research has established the link between digital media exposure and body-image outcomes, the specific relationship between mukbang viewing and body image remains theoretically complex. Mukbang represents a multifaceted form of media content, and its psychological effects may not be unidirectional.

On the one hand, mukbang viewing may be associated with more positive body-image evaluations. To the extent that individuals perceive mukbang as more influential on others than on themselves, a self-enhancement mechanism linked to third-person perception (Davison, 1983; Perloff, 2002) may foster the perception that they are less susceptible to media influence. Such perceptions can reinforce a sense of personal control and contribute to more favorable self-evaluations (Tal-Or et al., 2010).

On the other hand, mukbang viewing may also have negative implications under certain conditions. Drawing on social comparison theory (Festinger, 1954), exposure to media content may trigger upward comparisons, particularly when viewers evaluate their own eating behaviors or body-related characteristics against perceived norms. Empirical research has shown that exposure to appearance-related content on digital media platforms is associated with increased body dissatisfaction (Fardouly and Vartanian, 2016; Holland and Tiggemann, 2016). Similarly, from the perspective of self-discrepancy theory (Higgins, 1987), mukbang content may highlight discrepancies between individuals’ actual behaviors and their ideal or socially expected standards, potentially leading to negative self-evaluations.

Taken together, these contrasting findings suggest that the association between mukbang viewing and body-image evaluations depends on how viewers cognitively interpret media influence. Because mukbang is an entertainment-oriented genre that does not explicitly foreground appearance standards or persuasive appeals, and because prior work indicates that self-enhancement judgments consistent with third-person perception tend to dominate in non-persuasive, experiential media contexts (Jiang et al., 2024; Wang et al., 2025), the present study reasons that this indirect, discrepancy-based mechanism is more likely to characterize how young adults process mukbang content than direct appearance-based comparison. This reasoning guided the decision to examine perceptual gap, rather than comparison-based constructs, as the central mediating mechanism of the present study.

Although mukbang does not primarily emphasize physical appearance, its vivid sensory presentation and large-portion eating performances can evoke emotional reactions such as craving, satisfaction, or discomfort, activating self-reflective thoughts about one’s own eating behavior or body (Kircaburun et al., 2021a; Jiang et al., 2024). Experimental and observational evidence further shows that exposure to visually rich, food-centered content can shape nutritional perceptions, eating-related cognitions, and body-related evaluations through cognitive appraisal processes, even without explicit appearance cues (Kim et al., 2022; Song et al., 2025). Collectively, these findings suggest that mukbang may shape body-related cognitions primarily through indirect cognitive appraisal rather than direct persuasive influence (Li and Zhang, 2023).

One influential framework for understanding such indirect and discrepancy-based perceptions is the Third-Person Effect (TPE). First introduced by Davison (1983), TPE proposes that individuals tend to perceive media messages as exerting stronger influence on others than on themselves. This perceptual bias has been widely documented across diverse media contexts (Gunther, 1995; Eveland et al., 1999). Recent studies further indicate that TPE remains highly relevant in contemporary digital environments characterized by personalized and emotionally engaging content. Evidence from digital food-related contexts shows that viewers often report emotional and vicarious experiences while simultaneously perceiving others as more susceptible to media influence (Jiang et al., 2024; Wang et al., 2025).

Such patterns indicate that media effects can operate through indirect, experiential processes rather than explicit persuasion. Importantly, this suggests that third-person perception is not limited to traditionally persuasive or informational messages but can also arise in experiential and entertainment-oriented media contexts, thereby extending the theoretical scope of TPE.

Beyond establishing the presence of third-person perception, it is critical to examine its psychological implications for self-evaluation. Prior research suggests that perceiving others as more susceptible to media influence can serve a self-enhancement or defensive function, allowing individuals to maintain a favorable self-concept (Perloff, 2002; Tal-Or et al., 2010).

In the context of mukbang consumption, this perceptual discrepancy indicates that individuals tend to view themselves as relatively resistant to media influence, particularly with respect to eating behaviors and body-related cues embedded in food-related content. Such perceptions can reinforce a sense of personal control and self-regulatory capacity, both of which are closely associated with more positive self-evaluations.

From this perspective, perceptual gap can be conceptualized as a partial mediator linking media exposure to body-image evaluations. Specifically, exposure to mukbang content may shape individuals’ perceptions of media influence on the self versus others, which in turn informs their body-image evaluations. However, the strength and implications of this mediating process may not be uniform but instead depend on how individuals cognitively construe others in relation to themselves. This highlights the role of social distance in shaping how such self–other comparisons are constructed.

Social distance is an important factor shaping both third-person perception and the implications of perceptual discrepancy for self-evaluation. Prior research indicates that individuals differentiate media influence judgments depending on the perceived social proximity of the target group, tending to perceive greater media influence on socially distant others than on socially proximate groups (Perloff, 1999, 2008). This differentiation implies that the magnitude and implications of perceptual discrepancy vary depending on how “others” are socially constructed: judgments about distant groups tend to rely on generalized assumptions, whereas judgments about proximate groups involve more self-referential processing.

In this context, age-based groups provide a meaningful basis for social distance, as individuals may perceive peers, younger individuals, and older adults as differing in their susceptibility to media influence. These perceived differences in social proximity shape how individuals engage in self–other comparisons when forming self-evaluations, relying more heavily on comparisons with proximate targets while making more generalized judgments about distant others (Festinger, 1954; Wood, 1989; Perloff, 1999, 2008). Social distance is therefore conceptualized as a moderator of the relationship between perceptual gap and body-image evaluations.

Perceptual gap and social distance were selected as the focal constructs of the present study because they represent, respectively, the core mediating and moderating mechanisms most consistently identified in the third-person perception literature: perceptual gap operationalizes the self–other discrepancy that defines third-person perception itself (Davison, 1983), and social distance is the most robust and widely replicated moderator of that discrepancy, commonly referred to as the social distance corollary (Perloff, 1999, 2008). Other mechanisms discussed in the mukbang literature, such as parasocial engagement with content creators or general media literacy, were not incorporated as focal variables because they characterize viewers’ relationship with content or with media in general rather than the self–other evaluative comparison that is central to third-person perception; these constructs are instead treated as candidate boundary conditions for future research.

Despite these theoretical developments, important gaps remain in the existing literature. Although prior studies have documented the emotional, parasocial, and appetite-related dimensions of mukbang consumption, limited attention has been paid to how viewers cognitively evaluate mukbang’s influence on themselves versus others. In particular, little is known about whether such perceptual discrepancies are associated with body-image evaluations, or whether this relationship varies depending on social distance. These gaps are especially important in the case of Chinese young adults, who are highly exposed to mukbang content within an algorithm-driven and highly commercialized digital media environment.

Accordingly, the present study examines mukbang viewing among young adults in China and investigates third-person perception by comparing perceived media influence on the self and others. It further tests whether perceptual gap mediates the relationship between media exposure and body-image evaluations, and whether this effect is moderated by social distance. In doing so, the study aims to clarify the cognitive mechanisms through which digital food content shapes body-related perceptions.

### 1.1 Research hypotheses

Drawing on the theoretical framework and empirical evidence reviewed above, the following hypotheses are proposed.

*H1:* Mukbang viewing is positively associated with body-image evaluations.

*H2:* Perceptual gap mediates the association between mukbang viewing and body-image evaluations.

*H3:* Social distance moderates the positive association between perceptual gap and body-image evaluations, such that this association is stronger at higher levels of perceived social distance.

## 2 Materials and methods

### 2.1 Procedure

Data were collected in February 2024 through an online questionnaire. After accessing the survey link, participants viewed an introductory page explaining the purpose of the study, confidentiality protections, and the voluntary nature of participation. Clicking the consent option indicated agreement to participate. The survey required approximately eight to ten minutes to complete and was administered in a fixed order that included demographic items, body image evaluations, mukbang viewing habits and motivations, perceptual gap items, and social distance assessments. All responses were anonymous, and participants were free to discontinue the survey at any time.

### 2.2 Sampling

A total of 610 individuals initially completed the online survey. To align with the study’s focus on young adults, participants outside the target age range (18–30 years) were excluded. Specifically, 69 respondents aged under 18, 76 aged 31–40, and 69 aged above 41 were removed. After applying the age eligibility criterion, 396 responses were retained for the final analysis, representing 64.9% of the initial sample.

The questionnaire was administered through Wenjuanxing, a widely used online data collection platform in China. Participants were recruited using a non-probability, accessibility-based sampling strategy through university bulletin boards, WeChat groups, social media posts, and online community networks. Recruitment announcements briefly described the purpose of the study, and participation was entirely voluntary.

Before beginning the questionnaire, all respondents read an electronic informed consent statement describing the purpose of the study, confidentiality protections, and the voluntary nature of participation. Consent was provided by clicking the agreement button on the introductory page. No personal identifiers were collected, and all responses were anonymous.

Because the present study focused on young adults who constitute the demographic group most actively engaged with short-form video platforms and mukbang content in China, only individuals aged 18 to 30 were eligible for inclusion. Age was measured using predefined categories, and respondents selecting the 18–24 or 25–30 category were retained.

Among the final sample, 53.0 percent were male (*n* = 210) and 47.0 percent were female (*n* = 186). Most participants were undergraduate or graduate students, which aligns with demographic patterns typically observed among internet-using young adults in China. Educational level and other demographic characteristics were collected and included as control variables in subsequent analyses.

To ensure data quality, several exclusion procedures were subsequently applied to the 396 age-eligible responses. Following established survey-methodology guidance for detecting inattentive responding (Greszki et al., 2015; Leiner, 2019), responses completed in less than one-third of the median completion time were classified as excessively fast and flagged for exclusion. No respondents (0/396) fell below this threshold. Cases showing satisficing patterns, such as identical responses across all scale items or logically inconsistent answers, and surveys containing substantial missing data were screened using the same procedure; none of the 396 responses met these exclusion criteria. Accordingly, all 396 age-eligible responses satisfied the established data-quality criteria and constituted the final analytic sample.

All study procedures adhered to standard ethical practices for research involving human participants, and informed consent was obtained from all participants prior to data collection.

### 2.3 Measurements

#### 2.3.1 Body image

Body image was measured using six items adapted from the Body Image States Scale (BISS), which assesses momentary evaluations of one’s appearance and physical attributes (Cash et al., 2002). The items captured satisfaction with appearance, body shape, weight, attractiveness, and perceptions of looking better than usual and better than most people. Participants responded on a nine-point Likert scale ranging from one, meaning very dissatisfied, to nine, meaning very satisfied. All items followed the same response direction, so no reverse scoring was required. Higher scores reflected more positive body image. A mean score of the six items was computed to represent participants’ overall body image evaluation (hereafter referred to as body image). The scale demonstrated strong internal consistency in the present sample (Cronbach’s *α* = 0.85).

#### 2.3.2 Mukbang viewing habits

Mukbang viewing was assessed using four behavioral indicators that capture participants’ overall engagement with mukbang content: viewing frequency, average viewing duration, typical viewing time of day, and viewing motivations.

Viewing frequency was measured by asking participants how often they watch mukbang content on short-form video platforms. Viewing duration captured the typical length of time spent watching mukbang videos during a single viewing session. Viewing time of day was measured by asking respondents to indicate the period of the day during which they most frequently watched mukbang content.

Viewing motivations were assessed using nine items capturing common motivations for watching mukbang content (Kircaburun et al., 2021b). These items captured common motivations such as entertainment, relaxation, curiosity about food, and vicarious eating experiences. Responses were recorded on a five-point Likert scale ranging from 1 (strongly disagree) to 5 (strongly agree). The motivation scale demonstrated acceptable internal consistency in the present sample (Cronbach’s *α* = 0.77).

To capture overall engagement with mukbang content, the four behavioral indicators were standardized and averaged to create a composite index representing general mukbang viewing intensity. Standardization was applied to account for differences in the original response scales across the four indicators. Higher values indicate greater engagement with mukbang content.

#### 2.3.3 Perceptual gap

Perceptual gap was assessed using five items measuring participants’ perceived influence of mukbang content on different social targets, grounded in third-person perception theory. Participants rated the influence of mukbang on themselves and on four other groups: people in general, peers of similar age, younger individuals, and older adults. Responses were recorded on a five-point Likert scale ranging from 1 (very slight influence) to 5 (very strong influence). This measurement approach follows established operationalizations of third-person perception in media effects research (Choi et al., 2008; Li, 2008).

Following standard operationalizations in third-person perception research, the perceptual gap score was calculated as the difference between the mean perceived influence on the four “other” targets and the perceived influence on the self (others − self). Higher scores indicate a larger perceived self–other discrepancy in media influence.

#### 2.3.4 Social distance

Social distance refers to the perceived psychological or demographic distance between the self and different social groups when estimating media influence. In third-person perception research, individuals often evaluate media effects relative to socially proximate or distant groups, such as peers versus more socially distant populations (Perloff, 1999, 2008). Age-based reference groups are commonly used in third-person perception research because age differences provide a meaningful indicator of social proximity or distance when individuals estimate media influence on others. In the context of mukbang consumption, age-based reference groups provide a meaningful form of demographic distance that may shape how individuals estimate media influence on others.

Based on this theoretical perspective, social distance in the present study was operationalized using three age-based reference groups: peers of similar age, younger individuals, and older adults. Participants rated the perceived influence of mukbang content on each of these groups using a five-point Likert scale ranging from 1 (very slight influence) to 5 (very strong influence).

To clarify the relationship between the two measures, three of the social distance items (peers of similar age, younger individuals, and older adults) correspond to three of the four “other” targets used in the perceptual gap measure. The perceptual gap measure additionally included the category “people in general,” which was not part of the social distance measure. While the perceptual gap captures the overall discrepancy between perceived influence on the self and others, the social distance measure focuses specifically on differences in perceived influence across age-based “other” groups.

A mean score across the three items was computed to represent perceived social distance. The scale demonstrated acceptable internal consistency in the present sample (Cronbach’s *α* = 0.75).

Age, gender, and educational level were included as control variables to account for demographic differences that may influence media use and body-image-related responses. These variables were selected because prior research has shown that demographic characteristics are associated with both media consumption patterns and body image perceptions (see Table 1).

**Table 1:** Measurement summary of key constructs.

Construct	Items (brief)	Response format	Reliability
Body image	6 items on appearance satisfaction	9-point Likert scale	α = 0.85
Mukbang viewing habits	Duration, frequency, time of day; motivation scale (9 items)	Categorical + 5-point Likert scale	α = 0.77
Perceptual gap	5 items: influence on self vs. others	5-point Likert scale	—
Social distance	3 items: influence on age-based groups	5-point Likert scale	α = 0.75
Controls	Age, gender, education	—	—

Reliability coefficients are reported only for multi-item scales.

### 2.4 Statistical analysis

All statistical analyses were conducted using SPSS version 27. Descriptive statistics were computed to summarize participants’ demographic characteristics and to examine the distributions and central tendencies of all key variables. Internal consistency of multi-item scales was evaluated using Cronbach’s alpha coefficients.

Pearson correlation analyses were performed to examine bivariate associations among mukbang viewing indicators (including viewing motivations), perceptual gap, social distance, and body image evaluations. Following standard operationalizations in third-person perception research, perceptual gap scores were calculated by subtracting participants’ perceived influence of mukbang on the self from their perceived influence on others, with the latter operationalized as the mean perceived influence across the relevant target groups.

Hierarchical linear regression analyses were conducted to examine the direct and indirect associations among mukbang viewing, perceptual gap, and body image evaluations. Demographic variables, including age, gender, and educational level, were entered as control variables in the first step. Mukbang viewing and perceptual gap were entered in subsequent steps to assess their unique contributions to body image outcomes. For moderation analyses, social distance was included as a moderator by adding the interaction term between perceptual gap and social distance. Variables involved in interaction terms were mean-centered prior to analysis.

Mediation analyses were conducted using regression-based, bias-corrected bootstrapping procedures with 5,000 resamples to test indirect effects of mukbang viewing on body image evaluations via perceptual gap. For all analyses, statistical significance was evaluated using two-tailed tests with an alpha level of 0.05, and 95% confidence intervals were reported where applicable.

To evaluate whether the achieved sample size provided adequate statistical power, a post-hoc power analysis was conducted using G*Power 3.1.9.6 (Faul et al., 2009), F-test family, linear multiple regression. For the full direct-effect model (four predictors: age, gender, education, and mukbang viewing; *N* = 396), the observed effect size was large (Cohen’s f^2^ = 0.43, based on R^2^ = 0.301), yielding an achieved power exceeding 0.999 at *α* = 0.05. A sensitivity analysis further indicated that, with *N* = 396 and models containing up to seven predictors (the maximum used in the moderation analyses), the minimum effect size detectable with 80% power was approximately f^2^ = 0.02, corresponding to a conventionally “small” effect (Cohen, 1988). These results indicate that the sample size of 396 was sufficient to detect not only the observed large direct effect but also comparatively small effects in the mediation and moderation models, supporting the statistical robustness of the reported findings.

## 3 Results

### 3.1 Descriptive statistics and correlations

Descriptive statistics were computed to characterize the distributions, central tendencies, and variability of the main study variables. Mukbang viewing demonstrated variability across participants (M = 3.011, SD = 0.704), with observed scores ranging from 1.00 to 4.33, indicating differences in the frequency and intensity of participants’ engagement with mukbang content.

Body-image evaluations also exhibited substantial variability (M = 5.149, SD = 1.794). Scores ranged from 1.00 to 8.17, suggesting meaningful individual differences in participants’ evaluations of their physical appearance and body-related perceptions.

Perceptual gap scores were above the midpoint of the scale (M = 3.533, SD = 0.884), reflecting a tendency for participants to perceive mukbang content as exerting greater influence on others than on themselves. Social distance scores similarly indicated moderate levels of perceived psychological distance across age-based comparison groups (M = 3.685, SD = 0.923). Descriptive statistics and bivariate correlations among the main study variables are presented in Table 2.

**Table 2:** Descriptive statistics and correlations among the main study variables (**N* =* 396).

Variable	Mean	SD	1	2	3	4
Mukbang viewing	3.011	0.704	—			
Body image	5.149	1.794	0.549***	—		
Perceptual gap	3.533	0.884	0.494***	0.549***	—	
Social distance	3.685	0.923	0.049	0.220***	0.023	—

Higher scores indicate greater mukbang viewing intensity, larger perceptual discrepancy, more positive body-image evaluations, and greater perceived social distance. *p < 0.05, **p < 0.01, ***p < 0.001.

Pearson correlation analyses revealed several significant associations among the study variables. Mukbang viewing was positively associated with body-image evaluations (r = 0.549, *p* < 0.001) and perceptual gap (r = 0.494, *p* < 0.001), indicating that greater exposure to mukbang content was linked to more positive appearance evaluations and larger perceived discrepancies in media influence between oneself and others.

Perceptual gap was also positively associated with body-image evaluations (r = 0.549, *p* < 0.001), suggesting that both mukbang viewing and perceptual discrepancy were related to appearance-related self-evaluations at the bivariate level. In contrast, social distance showed a weaker but statistically significant association with body-image evaluations (r = 0.220, *p* < 0.001), while its associations with mukbang viewing (r = 0.049, *p* = 0.32) and perceptual gap (r = 0.023, *p* = 0.64) were not statistically significant.

### 3.2 Direct effect of mukbang viewing

Hierarchical regression analyses were conducted to examine the direct association between mukbang viewing and body-image evaluations. In the first step of the model, demographic variables including age, gender, and educational level were entered as control variables to account for background characteristics that may influence body-image outcomes. Mukbang viewing was added in the second step to assess its contribution beyond these demographic factors.

Results indicated that mukbang viewing was a significant predictor of body-image evaluations (B = 1.398, *p* < 0.001). The inclusion of mukbang viewing substantially increased the proportion of explained variance in body-image outcomes, with the full model accounting for 30.1% of the variance (R^2^ = 0.301). These findings indicate a robust association between mukbang viewing and body-image evaluations after controlling for demographic characteristics. The results of the direct effect model are presented in Table 3.

**Table 3:** Regression results for the direct effect model.

Predictor	B	*p*
Intercept	0.939	—
Mukbang viewing	1.398	< 0.001

Model summary: R^2^ = 0.301. Demographic variables (age, gender, educational level) were included as controls in Step 1.

### 3.3 Mediating role of perceptual gap

To examine whether perceptual gap functioned as a mediating mechanism linking mukbang viewing to body-image evaluations, mediation analyses were conducted using bias-corrected bootstrapping procedures with 5,000 resamples. Regression coefficients for all paths in the mediation model are summarized in Table 4.

**Table 4:** Mediation analysis results for mukbang viewing, perceptual gap, and body-image evaluations.

Path	B	*p*	95% CI
a: Mukbang → Perceptual gap	0.620	< 0.001	
b: Perceptual gap → Body image	0.747	< 0.001	
c (total effect): Mukbang → Body image	1.398	< 0.001	
c′ (direct effect)	0.935	< 0.001	
Indirect effect (a × b)	0.463	—	[0.431, 0.490]

Indirect effects were estimated using bias-corrected bootstrapping with 5,000 resamples. The 95% confidence interval did not include zero, indicating a statistically significant indirect effect.

Mukbang viewing significantly predicted perceptual gap (B = 0.620, *p* < 0.001), indicating that higher levels of mukbang exposure were associated with greater perceived influence on others relative to oneself. Perceptual gap also significantly predicted body-image evaluations when controlling for mukbang viewing (B = 0.747, *p* < 0.001).

When perceptual gap was included in the model, the direct effect of mukbang viewing on body-image evaluations remained significant (B = 0.935, *p* < 0.001). Compared with the total effect estimate (B = 1.398), the coefficient for mukbang viewing was reduced, indicating partial mediation. The bootstrapped indirect effect was statistically significant (a × b = 0.463), and the corresponding 95% confidence interval did not include zero ([0.431, 0.490]). These results indicate the presence of a reliable indirect pathway from mukbang viewing to body-image evaluations through perceptual gap.

### 3.4 Moderating role of social distance

To test whether social distance moderated the association between perceptual gap and body-image evaluations, perceptual gap and social distance were entered simultaneously into a regression model. Perceptual gap was positively associated with body-image evaluations (B = 0.747, *p* < 0.001), and social distance also showed a positive association (B = 0.102, *p* = 0.001).

An interaction term between perceptual gap and social distance was then included to test the moderation hypothesis. The interaction effect was not statistically significant (B = 0.014, *p* = 0.587), indicating that the association between perceptual gap and body-image evaluations did not vary across levels of social distance. Results of the moderation analysis are presented in Table 5.

**Table 5:** Moderation analysis results.

Predictor	B	*p*
Perceptual gap	0.747	<0.001
Social distance	0.102	0.001
Interaction (PG × SD)	0.014	0.587

**Figure 1:**
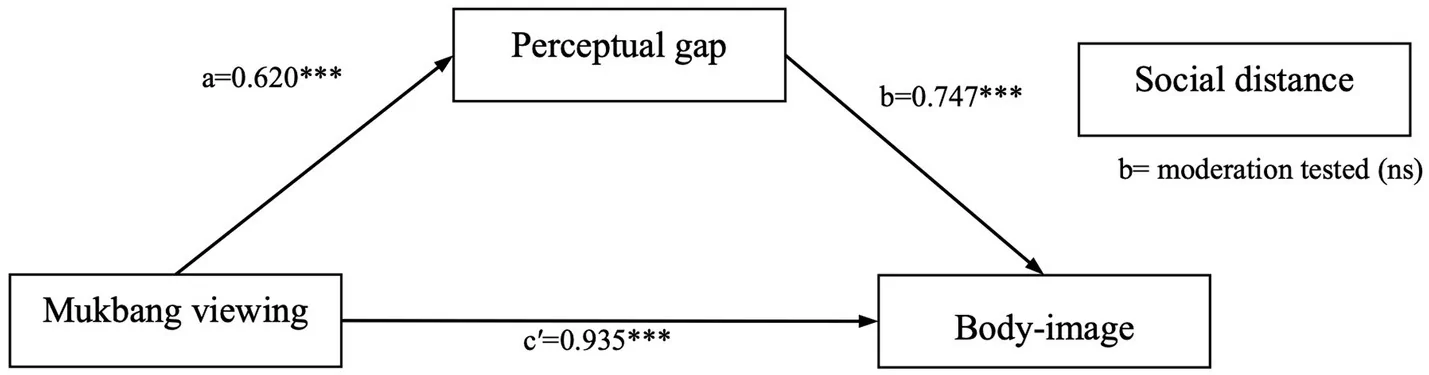
Perceptual gap was tested as a mediator between mukbang viewing and body-image evaluations, whereas social distance was examined as a moderator of the relationship between perceptual gap and body-image evaluations.

Conditional indirect effects were subsequently estimated to examine whether the indirect effect of mukbang viewing on body-image evaluations through perceptual gap differed by social distance. Indirect effects were calculated at one standard deviation below the mean, at the mean, and one standard deviation above the mean of social distance. The indirect effect remained significant and nearly identical across all three levels (low = 0.460; mean = 0.463; high = 0.465), and all corresponding confidence intervals excluded zero. Figure 1 provides a visual summary of the tested mediation and moderation model.

## 4 Conclusion and discussion

### 4.1 Discussion

The present study found that mukbang viewing was positively associated with body-image evaluations and that this relationship was partially mediated by perceptual gap. These findings suggest that young adults actively interpret digital food content rather than responding to it passively, consistent with recent work characterizing digital food media as a space in which identity, affect, and self-presentation are dynamically negotiated (Chan et al., 2025).

It is important to acknowledge, however, that mukbang represents a complex media stimulus, and the present cross-sectional design cannot rule out the possibility that its psychological effects are not uniformly positive for all viewers. Because viewing motives, media literacy, and individual differences in the relationship with food and body image were not directly assessed in this study, we did not test whether these factors alter the direction of the association. Rather, we identify them as plausible boundary conditions that future research should examine directly.

The finding that participants perceived greater media influence on others than on themselves indicates that third-person judgments emerge even for mukbang, a genre that is neither persuasive nor explicitly appearance oriented, extending such judgments to content outside the persuasive or appearance-focused media typically studied in this literature. This pattern aligns with research on digital commensality and technology-mediated eating practices, which suggests that everyday food-related media can elicit reflective and evaluative processing even without explicit persuasive intent (Spence et al., 2019), indicating that cognitive appraisal can occur during routine, low-involvement media consumption.

The emergence of perceptual discrepancy in this context indicates that third-person effects are not confined to political or harmful messages but also extend to entertainment-oriented media (Sun et al., 2008). This interpretation is consistent with experimental evidence reported by Tal-Or et al. (2010), suggesting that third-person perception functions not only as a perceptual bias but also as a broader cognitive mechanism underlying self-evaluative processes.

This mechanism is consistent with social comparison theory (Festinger, 1954), as self-evaluations are often constructed through implicit contrasts with others (Vogel et al., 2014). Exposure to food-related or lifestyle imagery can activate such comparative self-appraisal processes even in the absence of explicit appearance cues (Chae, 2018), suggesting that perceiving others as more susceptible to media influence functions as an implicit affirmation of one’s own self-regulatory capacity.

The analysis of social distance further refines theoretical expectations. Although third-person perception research has traditionally emphasized the role of social distance in amplifying self–other differentiation (Perloff, 2008), the present study found no evidence of such moderation. One interpretation is that demographic distance may be less salient in the context of mukbang, which is widely consumed across age groups and lacks strong generational boundaries. An alternative explanation is that mukbang content primarily emphasizes sensory and experiential aspects of eating rather than normative behavioral cues. Because the content does not explicitly promote particular lifestyles or appearance standards, viewers may be less inclined to differentiate media influence across specific social groups. Instead, they may rely on generalized assumptions about “others” when estimating media influence. This interpretation is consistent with arguments that third-person effects can occur even when social categories have limited salience (Chung and Moon, 2016).

Finally, the findings contribute to broader discussions regarding the psychological significance of digital food media. While prior research on mukbang has primarily emphasized viewing motivations, social connection, and problematic usage patterns, the present study highlights the cognitive landscape through which food-related media are interpreted. Even ostensibly non-persuasive digital genres appear capable of evoking evaluative judgments related to self-understanding and social comparison. Experimental research has shown that food-related digital imagery can activate self-referential evaluations even when attention is not explicitly directed toward appearance (Song et al., 2025). In addition, qualitative research on online mukbang communities indicates that mukbang environments are structured by shared norms and meanings related to eating, self-control, and bodily awareness, even when appearance is not explicitly emphasized (Strand and Gustafsson, 2020).

Taken together, these findings underscore the importance of examining how digital food practices shape cognitive appraisals, body-image perceptions, and self-evaluative processes among Chinese young adults, highlighting perceptual discrepancy as a key cognitive mechanism linking media exposure to body-image evaluations.

These findings should be interpreted with caution. First, the study relied primarily on a sample of university-affiliated young adults, which may limit the generalizability of the findings to the broader population of Chinese individuals aged 18–30. Second, the cross-sectional design precludes causal inferences regarding the relationships among mukbang viewing, perceptual gap, and body-image evaluations. Third, the use of self-reported measures may have introduced response bias.

#### 4.1.1 Contributions

First, the study addresses a significant conceptual gap in the mukbang literature, which has tended to emphasize motivations, affective gratifications, communal viewing experiences, or problematic consumption patterns, by identifying perceptual discrepancy as a meaningful cognitive process and extending the applicability of third-person perception (TPP) theory to this media domain. Classical TPP research has primarily focused on politically charged, risky, or morally sensitive messages; demonstrating that self–other influence judgments emerge during exposure to non-persuasive, entertainment-oriented content broadens the theoretical scope of TPP and supports the view that perceptual discrepancy constitutes a generalizable cognitive pattern across diverse media environments.

Second, the study contributes to integrative models linking cognitive appraisal processes with appearance-related outcomes. While body-image research has documented the role of social comparison, internalization and emotional processes, considerably less work has examined how individuals’ interpretations of media influence shape self-evaluative responses. The mediation findings clarify that perceptual discrepancy operates as a bridge between media exposure and body-image appraisal, offering a mechanism that complements existing affective and comparative explanations.

Third, the results refine theoretical assumptions regarding the role of social distance in TPP processes. Much of the literature posits that demographic or social distance amplifies self–other perceptual gaps. The absence of moderation in this study suggests that such assumptions may not generalize to entertainment contexts, particularly those consumed across heterogeneous audiences. This highlights the need for more context-sensitive formulations of TPP that consider media genre, audience diversity and content characteristics in addition to demographic distance.

Finally, the study contributes to emerging interdisciplinary discussions on digital food media by situating mukbang within broader communicative and psychological frameworks. The findings demonstrate that mukbang does not operate solely at the level of gratification or sociability but also engages viewers in interpretive processes relevant to identity, self-perception and the negotiation of bodily meaning. These findings broaden current understandings of digital food media by highlighting their cognitive and self-evaluative dimensions beyond entertainment and sociability.

#### 4.1.2 Practical implications

The findings provide practical insights into how young adults navigate digital food environments. Although mukbang is not explicitly oriented toward appearance, its association with body-image evaluations suggests that food-related entertainment may carry psychological meaning beyond its surface content. Digital food media may therefore be considered part of the broader environment in which young adults form, monitor, and adjust evaluations of their bodies.

A second implication relates to how viewers reason about media influence. The strong role of perceptual discrepancy indicates that young adults draw inferences about how mukbang affects “others,” and that these inferences are linked to their own appearance evaluations. Media-literacy initiatives may benefit from attending to these interpretive processes, not only to the content itself. Helping viewers recognize how judgments about others’ susceptibility shape their self-perceptions may support a more reflective understanding of online media.

These implications also extend to public-health communication. Mukbang commonly features stylized eating practices, including unusually large portions or rapid consumption. Although behavioral outcomes were not assessed in this study, the observed cognitive associations imply that such portrayals may contribute to perceptions of normative eating behavior. Public-health efforts targeting young adults may therefore benefit from addressing how digital food content is interpreted, alongside traditional nutritional messaging.

#### 4.1.3 Future studies

Future research may extend the present findings in several directions.

First, although the current study focused on young adults aged 18 to 30 who are highly engaged with mukbang content, this sampling strategy limits the generalizability of the findings. Including adolescents, older adults, and individuals from different cultural or platform contexts would help assess whether the cognitive pathways identified here operate similarly across populations with distinct media habits and body-image norms, thereby strengthening external validity.

Second, the present study relied on self-report measures capturing momentary cognitive and evaluative responses. While this approach is appropriate for identifying perceptual mechanisms, future research may benefit from incorporating mixed-method designs. Qualitative approaches, such as in-depth interviews or cognitive elicitation tasks, could provide deeper insight into how viewers reason about others’ susceptibility to media influence and how these interpretations are articulated in everyday contexts. Such methods would complement survey-based analyses by capturing dimensions of perceptual discrepancy that are not easily quantified.

Third, future studies may adopt experimental designs to examine how specific features of mukbang content evoke perceptual discrepancy. Manipulating elements such as portion size, eating pace, or creator persona would allow researchers to identify which aspects of digital food entertainment are most salient in shaping influence judgments and appearance-related evaluations.

Finally, examining links between cognitive appraisals and behavioral or physiological outcomes represents an important direction for future work. Integrating measures of eating behavior, emotional responses, or health-related practices would help clarify how interpretations of mukbang extend beyond self-evaluations to influence broader aspects of well-being. Future research may also examine how platform-specific cultures and regional digital media environments shape the interpretation and impact of mukbang content, thereby clarifying the behavioral and contextual implications of digital food media exposure.

## 5 Conclusion

This study examined the associations between mukbang viewing, perceptual gap, and body-image evaluations among 396 Chinese young adults through a Third-Person Effect framework. Mukbang viewing was positively associated with body-image evaluations, and this association was partially mediated by perceptual gap, whereas social distance did not moderate the relationship between perceptual gap and body-image evaluations. These findings suggest that perceptual discrepancy functions as a key cognitive mechanism linking entertainment-oriented digital food content to body-image evaluations, extending the application of the Third-Person Effect framework to mukbang viewing.

## Data Availability

The raw data supporting the conclusions of this article will be made available by the authors, without undue reservation.
